# LCK as a Potential Therapeutic Target for Acute Rejection after Kidney Transplantation: A Bioinformatics Clue

**DOI:** 10.1155/2018/6451298

**Published:** 2018-06-07

**Authors:** Linpei Jia, Rufu Jia, Yinping Li, Xiaoxia Li, Qiang Jia, Hongliang Zhang

**Affiliations:** ^1^Department of Nephrology, Xuanwu Hospital of Capital Medical University, Changchun Street 45#, Beijing 100053, China; ^2^Central Hospital of Cangzhou, Xinhua Middle Street 201#, Cangzhou, Hebei Province 061001, China; ^3^Department of Life Sciences, The National Natural Science Foundation of China, Shuangqing Road 83#, Beijing 100085, China

## Abstract

**Objectives:**

We aim to identify the key biomarker of acute rejection (AR) after kidney transplantation via bioinformatics methods.

**Methods:**

The gene expression data *GSE75693* of 30 samples with stable kidney transplantation recipients and 15 AR samples were downloaded and analyzed by the limma package to identify differentially expressed genes (DEGs). Then, Gene Ontology (GO) functional enrichment analysis and Kyoto Encyclopedia of Genes and Genomes (KEGG) pathway analysis were done to explore the biological functions and potential important pathways of DEGs. Finally, protein-protein interactions (PPIs) and literature mining were applied to construct the cocitation network and to select the hub protein.

**Results:**

A total of 437 upregulated genes and 353 downregulated genes were selected according to *P* < 0.01 and |log_2_(fold change)| > 1.0. DEGs of AR are mainly located on membranes and impact the activation of receptors in immune responses. In the PPI network, Src kinase, lymphocyte kinase (LCK), CD3G, B2M, interferon-*γ*, CD3D, tumor necrosis factor, VAV1, and CD3E in the T cell receptor signaling pathway were selected as important factors, and LCK was identified as the hub protein.

**Conclusion:**

LCK, via acting on T-cell receptor, might be a potential therapeutic target for AR after kidney transplantation.

## 1. Introduction

Kidney transplantation is widely accepted as a standard life-saving therapy for end-stage chronic kidney disease [1]. Frequently as the last resort, kidney transplantation may prolong patients' survival and improve their life quality [2]. Immunosuppressants are routinely prescribed to recipients to promote graft survival after transplantation [3]. Despite the standard use of immunosuppressants, however, acute rejection (AR) is sometimes inevitable, which usually occurs days to months after transplantation [4]. The short- and long-term impacts of AR on graft loss and even morbidity have been extensively studied [5].

According to the distinct key players in the pathogenesis, AR is classified into two types, i.e., acute T cell-mediated rejection (TCMR) and acute antibody-mediated rejection (AMR). Although the precise mechanism of TCMR is still unclear, cell-mediated cytotoxicity of parenchymal cells and local cytokine release are two possible causes [6]. By contrast, AMR is caused by circulating antibodies of recipients, which are mainly antibodies against donor human leukocyte antigen (HLA). Then T helper (Th) cell and macrophages participate in the immune response to clear allogenic cells and to assist differentiation of B cells [7]. Thus, T cells play important roles in both TCMR and AMR.

Once AR occurs, a short course of intensive immunomodulation is needed. Pulse steroid therapy, alteration of immunosuppressants, monoclonal antibodies and combinations thereof are common therapies for AR [8]. Among others, a high dose of steroids and immunosuppressants might lead to a high risk of infection and other side effects [9]. In recent decades, monoclonal antibodies, such as antilymphocyte globulin, antithymocyte globulin, and T10B9, an monoclonal antibody against the T cell receptor (TCR) [10], have been applied to deal with AR [9]. However, side effects such as infection and tumorigenesis may still occur occasionally [11]. Webster et al. compared the efficacy between monoclonal antibodies and steroids; they found that monoclonal antibodies appeared better than steroids for reversing first acute cellular rejection and preventing graft loss, but there was little or no difference in subsequent rejection and the survival rate [9]. Thus, further insight into the mechanism of AR and identification of the key step in the pathogenesis may shed light on the discovery of therapeutic targets for AR [12].

Gene expression analysis by bioinformatics methods has been widely used in genomics and biomedical studies, which helps clarify the molecular events underlying human biology and diseases [13]. Data mining of the available microarray could help narrow down the study scope so as to find research gaps [14]. In this study, we analyzed the public microarray data by using bioinformatics methods, including differentially expressed genes (DEGs) analysis, gene enrichment methods, protein-protein interaction (PPI) analysis, and literature miming, in order to identify the key factors of AR in kidney transplantation recipients and to provide new insights into the treatment of AR.

## 2. Materials and Methods

### 2.1. Affymetrix Microarray Data and Sample Selection

First, we downloaded the microarray dataset *GSE75693* from the public Gene Expression Omnibus (GEO) database (http://www.ncbi.nlm.nih.gov/geo/). The dataset *GSE75693* consists of the gene expression information of kidney biopsies from 30 stable renal allograft recipients, 15 patients with AR, 15 with BK virus nephropathy, and 12 with chronic allograft nephropathy. All subjects were pediatric and young adult recipients who received transplantation between 2000 and 2011 at the Lucile Packard Children's Hospital of Stanford University [15]. AR was defined at minimum, as per Banff schema, a tubulitis score ≥ 1 accompanied with an interstitial inflammation score ≥ 1. Normal allografts were considered as an absence of significant injury pathology of Banff schema [15]. Data of the 30 stable renal allograft recipients and the 15 patients with AR were used to investigate the potential mechanism of AR. In the original study, AR patients were diagnosed by professional pathologists and scored by the Banff and Chronic Allograft Damage Index as acute cellular or humoral rejection with clinical graft dysfunction and tubulitis and/or vasculitis on histology [15]. Stable renal transplant recipients were diagnosed as stable without histological or clinical graft injury [15].

The array data were based on the platform of GPL570 Affymetrix Human Gene U133 Plus 2.0 Array (Affymetrix Inc., Santa Clara, CA, USA). The raw data were preprocessed by Robust Multiarray Average [16] algorithmin affy package of Bioconductor (http://www.bioconductor.org/), including background correction, normalization, and calculation of gene expressions.

### 2.2. DEG Analysis

We used the limma package of Bioconductor to analyze DEGs between BKVN and nonallograft injury patients in R project (Supplementary 1). Linear models were constructed for gene expression data of AR and stable renal allograft samples, respectively. The contrast model was used to compare gene expression differences between the two groups. *P* values were calculated by the Bayesian *t*-test and adjusted by false discovery rate. DEGs were selected based on the threshold *P* < 0.01 and |log_2_(fold change)| > 1.0 [17]. The *P* value herein was used to test if the gene was differentially expressed between the AR and the stable groups with the |fold change| > 2.0.

### 2.3. Enrichment Analysis of DEGs

By Gene Ontology (GO) and the Kyoto Encyclopedia of Genes and Genomes (KEGG) in DEG enrichment analysis, we further investigated the potential mechanisms of AR. GO annotated genes by a defined, structured, and controlled vocabulary [18], including molecular function (MF), biological process (BP), and cellular components (CC), while KEGG assigns DEGs to specific pathways [19]. GO and KEGG can be performed in website of Database for Annotation, Visualization and Integrated Discovery (DAVID, http://david.abcc.ncifcrf.gov/). The potential GO annotation and pathways were selected based on *P* < 0.01 and count ≥ 5 [20].

### 2.4. PPI Network Construction

Connections and interaction networks of DEGs mean PPI. We uploaded the DEGs into the website of STRING (Search Tool for the Retrieval of Interacting Genes/Proteins, http://string-db.org/), which is the web source of biological database. According to the official explanation of STRING, the confidence score is the approximate probability that a predicted link exists between two proteins in the same metabolic map in the KEGG database (*Getting Started* in https://string-db.org/cgi/help.pl). Thus, PPIs of DEGs were selected with the threshold of score (high confidence) > 0.7 [20]. Then the analysis results were downloaded and modified by Cytoscape (http://www.cytoscape.org/). The proteins with higher degrees of interaction were considered as hub proteins [19].

### 2.5. Literature Mining

According to the analysis of STRING, hot proteins in the PPI were put into GenCLiP 2.0 (http://ci.smu.edu.cn/GenCLiP2.0/confirm_keywords.php), which is an online tool for literature mining of gene functions. In the website, biological keywords of hot proteins in previous literature database were analyzed by Gene Cluster with the Literature Profiles module with the threshold of *P* ≤ 1 × 10^−6^ and hits ≥ 6 [19]. And the Literature Mining Gene Networks module was used to show the cocitation network of hot proteins. After combining the results of literature mining and KEGG analysis, the primary pathways in AR were determined.

### 2.6. Hub Protein Selection by CytoNCA

In Cytoscape, the separated proteins from the network were removed. All nodes in PPIs were analyzed by CytoNCA. According to degree centrality, betweenness centrality, and subgraph centrality [16], the hub protein, which interacts most frequently with other proteins and works like a hub in the network, was selected. Finally, proteins associated with hub proteins at degree ≥ 20 were selected to construct the significant network about the mechanism of AR [21].

## 3. Results

### 3.1. Seven Hundred and Ninety DEGs Were Identified

**Figure 1:**
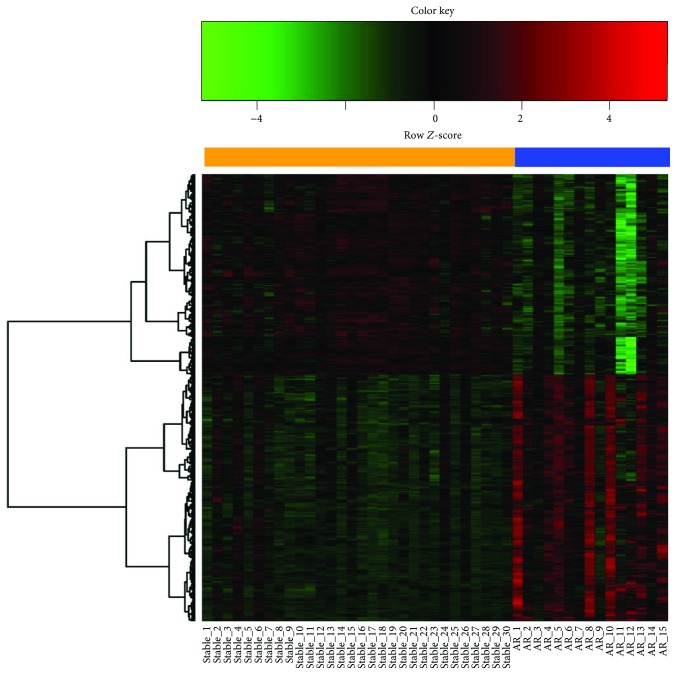
Heat map of differentially expressed genes. The row means a gene, and the column means a sample. Samples of stable allograft recipient are presented as the yellow bar, and samples of acute rejection (AR) patients are presented as the blue bar. Downregulated genes are shown in color green, while upregulated ones are in red. No difference expressed genes between AR and stable allograft patients are in black.

Microarray data in the *GSE75693* dataset were downloaded for further analysis. DEGs of AR were identified by the limma package following the process of linear model, contrast model, and DEGs selection. Compared with no-allograft injury patients, 790 genes are expressed differentially in AR patients based on the criteria of *P* < 0.01 and |log_2_(fold change)| > 1.0, including 437 upregulated genes and 353 downregulated genes. The hierarchical cluster analysis was done to show the distribution of DEGs (Figure 1).

### 3.2. DEGs of AR Mainly Enriched in the Cell Receptor Functions

**Figure 2:**
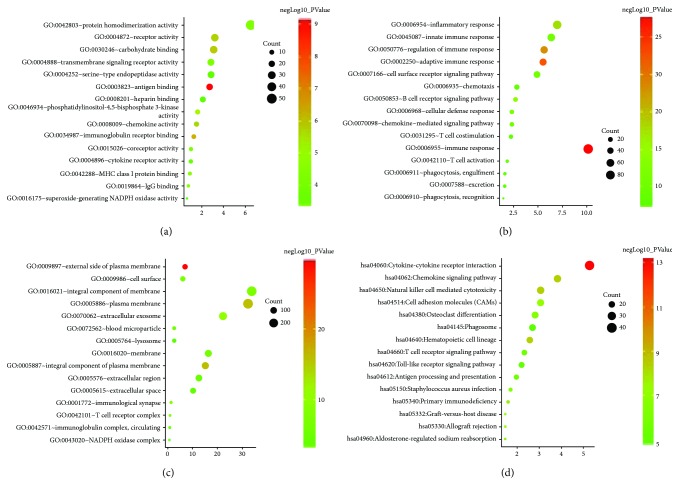
Bubble charts of top 15 gene enrichment results in each category. Gene counts and *P* values of top 15 enrichment terms in molecular function (MF), biological process (BP), cellular component (CC), and pathway analysis were shown in bubble charts. Gene counts in each term were displayed as the size of bubbles. Larger in size means larger numbers of genes in this term. The gradual color from red to green shows the changes of *P* values from low to high. (a) Results of MF analysis. Most of DEGs clustered in protein homodimerization activity, while the antigen binding has the greatest statistical significance. (b) Results of BP analysis. The immune response presented the less *P* value and the maximum of genes. (c) Results of CC analysis. Most of DEGs located on integral component of membrane, while the external side of plasma membrane showed the greatest significance in statistics. (d) Results of pathway analysis. Both the maximum of gene counts and lowest *P* value appeared in the category of cytokine-cytokine receptor interaction.

For further analyzing biological functions of DEGs, we uploaded DEGs in DAVID. GO and pathway terms were selected. In MF ontology, DEGs mainly enriched in 27 categories (Supplementary 2, Figure 2(a)) including the protein homodimerization activity (53 genes), receptor binding (26 genes), and receptor activity (26 genes). In BP ontology, 94 terms were identified, and the majority of enriched categories are the immune response (83 genes), signal transduction (81 genes), and the inflammatory response (57 genes), which focused on the immune process (Supplementary 3, Figure 2(b)). Distribution of DEGs on cells was shown in CC ontology. Proteins of DEGs were mostly located on membranes of cell or organelles, including integral component of membranes (275 genes) and plasma membranes (263 genes) (Supplementary 4, Figure 2(c)). Other important CC categories are the extracellular exosome (182 genes), extracellular region (102 genes), and the extracellular space (83 genes). In KEGG analysis, 23 potential pathways in AR are shown in Supplementary 5 and Figure 2(d). DEGs clustered in pathways of cytokine-cytokine receptor interaction (43 genes), chemokine signaling (31 genes), cell adhesion molecules (25 genes), and natural killer cell-mediated cytotoxicity (25 genes).

Enrichment analysis gives us a hint to infer the potential biological functions, locations, and participated pathways of DEGs. The presenting data demonstrate that DEGs of AR are most likely to be receptors or molecules interacting with receptors on the membrane to participate in a variety of immune responses.

### 3.3. TCR Signaling Transduction Is the Main Pathway in the Pathogenesis of AR

**Figure 3:**
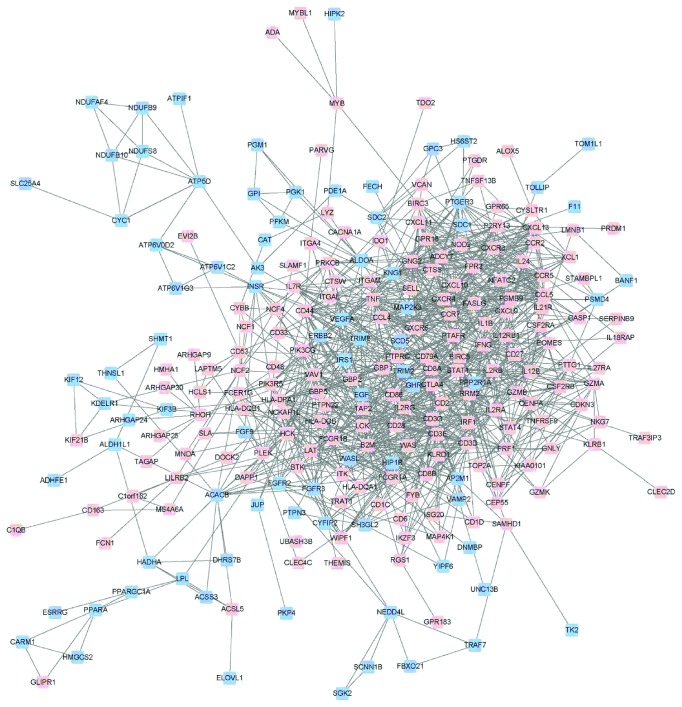
Protein-protein interaction network constructed with differentially expressed genes. Upregulated genes in AR were colored in red, while downregulated genes were in blue. A total of 254 DEGs were selected in the protein-protein interaction network, and DEGs were mainly focused on immune process.

**Figure 4:**
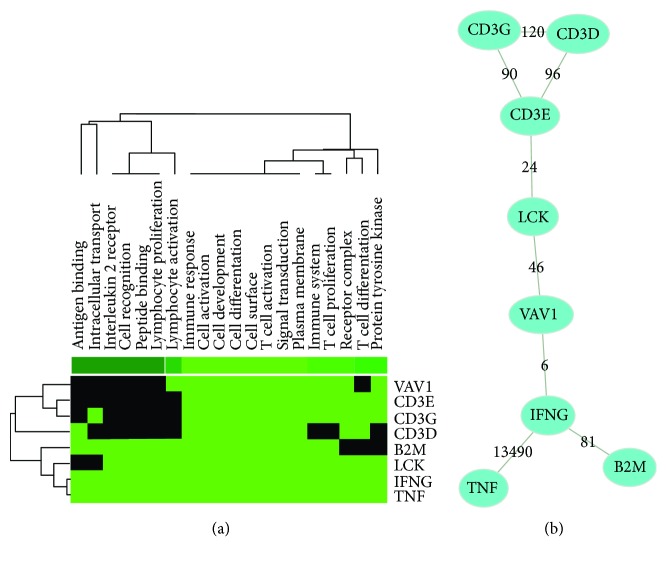
Literature mining results of top 8 differentially expressed proteins in degree. (a) Clustering analysis of *LCK*, *CD3G*, *B2M*, *IFNG*, *CD3D*, *TNF*, *VAV1*, and *CD3E* in previous studies. In the heat map, each row represents a gene, and each column represents a biological keyword. The color black means that the keyword has not been reported in this gene, and the color light green means that this keyword is related to this gene. Hot genes are mainly clustered in immune response, cell activation, cell differentiation, cell surface, T-cell activation, signal transduction, and plasma membrane. (b) Cocitation network of hot genes. In the cocitation network, all of the 8 genes were closely interacted. The numbers noted on the line indicate the number of studies cocited.

At first, 790 DEGs were uploaded in STRING website. Then 254 genes with score > 0.7 (high confidence) were selected to construct the PPI network by Cytoscape (Figure 3). Top eight hot genes, including *LCK*, *CD3G*, *B2M*, *IFNG*, *CD3D*, *TNF*, *VAV1*, and *CD3E*, were put into GenCLiP 2.0 for analysis of Gene Cluster with Literature Profiles and Literature Mining Gene Networks. Results of gene clustering indicated that keywords of hot genes reported in literature were immune response, cell activation, cell differentiation, cell surface, T-cell activation, signal transduction, and plasma membrane (Figure 4(a)). Information of the cocitation network was shown in Figure 4(b) and Table 1. *LCK* and *VAV1* work as links between *CD3E* and *IFNG*. Combined with pathway analyses, 7 hot genes, except for B2M, participate in the TCR signaling pathway, and *LCK*, *VAV1*, *IFNG*, and *TNF* participate in the natural killer cell-mediated cytotoxicity pathway.

### 3.4. LCK Is the Key Factor in AR

**Figure 5:**
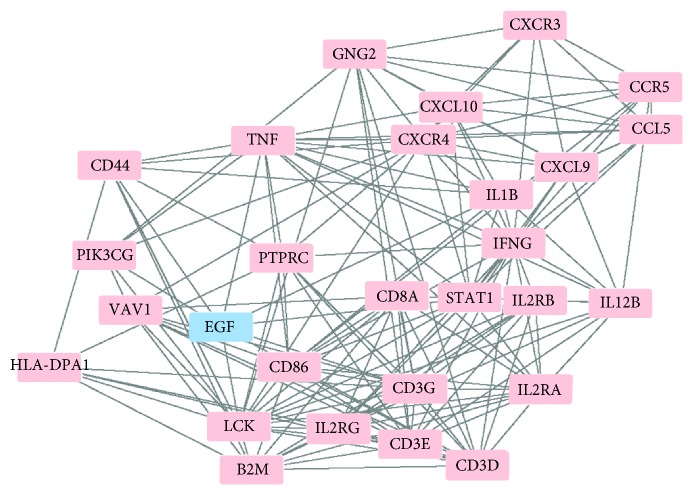
Protein-protein interaction (PPI) network of important proteins. Upregulated proteins are in red, and downregulated ones are in blue. The PPI network consists of 27 upregulated proteins including LCK, CD3G, CD3E, CD3D, IFNG, VAV1, TNF, B2M, CXCR3, CCR5, CCL5, CXCL9, CXCL10, CXCR4, GNG2, IL1B, IL12B, IL2RB, IL2RA, IL2RG, STAT1, CD8A, CD86, PTPRC, PIK2CG, CD44, and HLA-DPA1 and 1 downregulated protein EGF. LCK, CD3G, and IFNG are identified as hub proteins.

Each DEG was evaluated according to degree centrality, betweenness centrality, and subgraph centrality, respectively, in CytoNCA (Table 2). LCK ranked top 5 in all centralities indicating the important role of LCK in the pathogenesis of AR. The other hub proteins are CD3G and IFNG. Hub nodes and proteins directly associated with hubs constructed an interaction network (Figure 5), including 27 upregulated and 1 downregulated proteins. EGF is the only downregulated protein in the final network.

## 4. Discussion

In the present study, we aimed at investigating potential therapeutic targets for AR after kidney transplantation. First, 437 upregulated genes and 353 downregulated genes were selected as DEGs of AR compared with nonrejection recipients. According to gene enrichment, DEGs of AR are mainly located on membranes and participate in the TCR signaling pathway. We identified LCK as the potential key factor in the pathogenesis of AR.

By GO annotation in DAVID, we further explored the biological functions of DEGs. The results of functional annotations helped us infer the possible effects of DEGs on how AR occurs. According to results of BP, CC, and MF ontologies, the majority of DEGs were enriched in functions of membrane receptors in the immune process. AR in kidney transplantation is generally considered as an alloimmune response process involving networks of interacting cells throughout the body [22]. Our BP analysis showed that the mechanism of AR was primarily focused on immune process, and this finding is consistent with previous studies [6, 23]. Then the CC ontology indicated that most of DEGs were membrane structures, such as proteins on integral component of membrane and plasma membrane. Based on previous studies, AR may be mediated by cellular and/or humoral mechanisms [7]. Approximately 90% of AR is TCMR, which involves CD4^+^ and CD8^+^ T cells by activating perforin/granzyme degranulation pathways [24]. Regardless of the recognition of antigens in TCMR and AMR or the release of accessory molecules, such as cytokines, signaling molecules, and adhesion molecules, receptors on membrane play important roles [25]. Meanwhile, we also found that a majority of DEGs were involved in protein homodimerization in MF ontology. This indicated that protein homodimerization activity is the main structure transformation and activation of receptors [26] implicated in the antigen recognition and signaling transduction pathway in AR.

In the cocitation network, CD3D, CD3E, and CD3G, which are compositions of CD3 complex of TCR, interacted with each other to affect the assembly of TCR membrane complex and disturb T-cell responsiveness [27], especially CD3E [28]. After TCR engagement, the phosphorylation of CD3 immunoreceptor tyrosine-based activation motifs of CD3E in CD3 complex is combined with activated LCK, which is also called Src kinase lymphocyte kinase [29]. LCK is a member of protein tyrosine kinase involved in TCR signal transduction [30]. In TCR stimulation process, LCK can activate VAV GTPase to control the status of ezrin and moesin cytoskeletal protein phosphorylation, which regulates a series of protein translocation events [31, 32]. A previous study demonstrated that both AMR and TCMR manifested strong expressions of IFNG [33]. In AR, IFNG cooperated with TNF to increase antigen availability in rejection by enhancing HLA expression [34]. However, the relationship between VAV1 and IFNG has not been reported in the pathogenesis of AR, which could be further verified in animal and cellular experiments. Finally, via the PPI network analysis, we revealed that LCK in the TCR signaling pathway is the hub protein in the interaction network of DEGs, which underlies the notion that LCK might be a key link between antigen recognition and cytotoxicity.

The strength of our study is the combined use of several bioinformatics methods including DEG analysis, GO, KEGG, literature mining, STRING, and the PPI analysis. These data mining methods may corroborate each other and make the results reliable. However, our study has limitations. The sample size is relatively small. Different from conventional method, however, the limma method is proven effective in microarray analysis [35], even for a small sample size (2–5 cases) [36]. Nevertheless, the statistical power cannot be calculated in that the limma method comprises multistep data processing including both linear models and contrast models, as well as Bayesian analysis [37]. The data were downloaded from one dataset, and the sample size in GSE75693 was relatively small. The detailed demographic features of the cohort were unavailable in the published article. Due to the retrospective and bioinformatics nature of our study, further laboratory investigations on the cellular and animal levels are necessary to elucidate the pathogenesis of AR and to find potential therapeutic targets.

## 5. Conclusions

In summary, we revealed the potential important role of LCK in the pathogenesis of AR. LCK interacts with other 27 proteins and is actively involved in TCR pathway to activate the T cells in AR. LCK might be a potential therapeutic target for AR.

## Figures and Tables

**Table 1:** Hub genes identified by literature mining.

Gene	Cogenes (*n*)	Cocitations (*n*)	Total (*n*)
*IFNG*	3	13,563	68,347
*CD3E*	3	141	745
*VAV1*	2	52	822
*LCK*	2	68	2195
*CD3G*	2	145	327
*CD3D*	2	151	278
*TNF*	1	13,490	91,108
*B2M*	1	81	6479

**Table 2:** Top 5 genes evaluated by degree centrality, betweenness centrality, and subgraph centrality in the protein-protein interaction network.

Protein	Degree centrality	Protein	Betweenness centrality	Protein	Subgraph centrality
LCK	41.75	ACACB	20,963	LCK	4,376,067.5
CD3G	39.52	TNF	16,189	CXCR4	4,266,117
B2M	36.80	LCK	15,414	CD3G	3,693,030
IFNG	36.38	EGF	15,044	GNG2	3,554,691.25
CD3D	35.03	PIK3CG	10,487	IFNG	3,534,239.5

## Supplementary Materials

Supplementary Materials Supplementary 1: R codes of differentially expressed genes. Supplementary 2: results of molecular function analysis. Twenty-seven terms were selected according to *P* < 0.01 and count ≥ 5. Supplementary 3: results of biological process analysis. Ninety-four categories were selected according to *P* < 0.01 and count ≥ 5. Supplementary 4: results of cellular component analysis. Twenty-one categories were selected according to *P* < 0.01 and count ≥ 5. Supplementary 5: results of pathway analysis. Twenty-three pathways were selected according to *P* < 0.01 and count ≥ 5.
